# Tree co-occurrence and transcriptomic response to drought

**DOI:** 10.1038/s41467-017-02034-w

**Published:** 2017-12-08

**Authors:** Nathan G. Swenson, Yoshiko Iida, Robert Howe, Amy Wolf, María Natalia Umaña, Krittika Petprakob, Benjamin L. Turner, Keping Ma

**Affiliations:** 1 Department of Biology, University of Maryland, College Park, MD 20742 USA; 2 Kyushu Research Center, Forestry and Forest Products Research Institute, Kumamoto, 860-0862 Japan; 3 Department of Natural and Applied Sciences, University of Wisconsin-Green Bay, Green Bay, WI 54311 USA; 4 Smithsonian Tropical Research Institute, Apartado, 0843-03092 Balboa, Ancon Republic of Panama; 5 State Key Laboratory of Vegetation and Environmental Change, Institute of Botany, The Chinese Academy of Sciences, Beijing, 100093 China

## Abstract

The distribution and co-occurrence of species are partly the outcome of their interactions with environmental drivers. Drought is a key driver related to the distribution of plant species. Drought events continue to increase in frequency and severity and identifying those aspects of plant function that are related to drought is critical. Here, we perform a community-level analysis of gene expression in relation to experimental drought and relate the similarity in gene set enrichment across species to their natural co-occurrence. Species with similar gene set enrichment in response to experimental drought tend to non-randomly co-occur in a natural stand. We demonstrate that similarity in the transcriptomic response of species to drought is a significantly better indicator of natural co-occurrence than measures of functional trait similarity and phylogenetic relatedness and that transcriptomics has the capacity to greatly enhance ecological investigations of species distributions and community structure.

## Abstract (web summary)

Drought has a major influence on plant distribution. Here, Swenson et al. show that a similar gene expression response to experimental drought outperforms traditional functional traits and phylogenetic relatedness as a predictor of co-occurrence of tree species in a natural stand.

## Introduction

Environmental drivers interface with organismal function to determine individual demographic performance that scales up to partly determine species distributions through space and time^1^. Drought is known to be a key driver of tree species distributions and forest functioning from local to regional spatial scales^2,3^. Drought events have massive impacts on forest demography and ultimately carbon flux^4,5^. The strength and periodicity of drought events are expected to increase as regional climates undergo change^6^. In order to predict the effects of drought-induced change in forests, tree ecologists must first identify those aspects of plant function that best indicate drought response and to link those aspects with the distributions and demography of species in natural stands.

The way in which species respond to drought should influence their spatial co-occurrence in one of two ways. First, species with similar responses should tend to co-occur. This may be caused when less drought tolerant species exhibit superior performance in wetter environments, thereby excluding more drought tolerant species that will dominate drier environments. This mechanism may be manifested at both local and regional scales^2^. Alternatively, species with dissimilar strategies in response to drought (i.e., tolerators vs. avoiders) may be expected to co-occur at a higher frequency due to a reduction in direct competition during periods of low water resource availability. This mechanism may be more likely to occur in areas with strong seasonality in precipitation (i.e., deserts and tropical dry forests), compared with a seasonal rain forests or temperate deciduous forests.

There has been a marked increase in the number of studies that seek to elucidate the environmental drivers of species distributions and community structure^1,7–9^ by quantifying functional traits believed to be representative of the major axes of plant ecological strategy^10,11^. In concept, functional traits are traits directly related to the molecular functions or biological processes that impact performance or fitness^1^. However, in practice, typically only a few functional traits are measured and these traits are often only loosely or indirectly related to the physiological processes or rates of interest. In addition, these traits are representative of only a small portion of the diversity of functions in a plant^9^. A further limitation of functional trait-based ecology is that it generally assigns static trait values to individuals or species, thereby failing to capture the dynamic responses of an organism to important and temporally dynamic environmental drivers such as water availability.

In recognition of the limitations to the functional trait-based approach where important unmeasured traits and/or complex phenotypes such as response to drought are not well-estimated, some have proposed that phylogenetic information can be utilized as a way to estimate the unmeasured ecological similarity between species^12,13^. This approach, however, critically relies on an unknown degree of phylogenetic signal in function, and even in the best-case scenario it is a blind approach where the functional mechanisms driving the phylogenetic pattern will never be known. Combined, the above limitations may explain in part why functional trait and phylogenetically-based studies of plant distributions and community structure often uncover patterns that are weak or do not clearly point to a mechanism^7,14–16^. It is therefore likely that functional trait-based and phylogenetic investigations omit a great deal of mechanistic detail. Specifically, detail regarding how key environmental drivers such as drought and organismal function interface to determine the spatial distribution and co-occurrence of species.

Here, via a community-level analysis of gene expression in relation to experimental drought, we demonstrate that similarity in the transcriptomic response of species to drought is a significantly better indicator of natural co-occurrence than measures of functional trait similarity and phylogenetic relatedness.

## Results

### Transcriptomic response of seedlings to experimental drought

Here we quantified the dynamic physiological response of tree seedlings from 21 species to experimental drought through the investigation of transcriptomic data and asked whether gene expression to experimental drought was related to the co-occurrence of these species in a topographically heterogeneous long-term forest dynamics plot in northern Wisconsin, USA (Supplementary Table 1). The forest plot is 25.2 ha in area and we analyzed the 630 20 × 20 m subplots in the plot where all free-standing woody stems 1 cm or greater in diameter are tagged, mapped, measured, and identified. The plot has a well-characterized soil moisture gradient (Methods) with low elevations in the plot that have standing water during the late spring and early summer and higher elevations with more xeric soils. To test the perceived importance of this gradient and to integrate transcriptomic information into community ecology, we exposed 1-year old seedlings from these species collected from the region to experimental drought in the greenhouse and quantified differential gene expression between control and droughted plants for each species (Methods). We acknowledge that our forest plot data are from individuals far larger than seedlings and that this is a limitation of the study. However, we also note that previous work has demonstrated that differential demography at the seedling stage in response to abiotic and biotic conditions leaves a lasting imprint on juvenile and adult tree assemblages^2^. Next, for each of these species we quantified gene set enrichment, ignoring the directionality of the change in expression, for shared molecular function and biological process gene ontology (GO) terms identified across species. This approach enabled us to compare similar functions and processes across species without having to identify shared individual homologous genes across species. It is important to note that only GO categories identified across all species were used in this study to facilitate an unbiased comparison. A downside of this approach is that it relies on annotations derived from potentially distantly related model species, which could limit the scope of valid inferences^17^. Next, we then clustered species by their similarity in gene set enrichment across all molecular functions or biological processes (Supplementary Figs. 1, 2). This allowed us to generate a metric of overall similarity in expression similarity to drought across all species via branch lengths on the hierarchical clustering dendrogram. In sum, we were able to quantify the inter-specific similarity in gene set enrichment in response to experimental drought for GO biological processes and GO molecular functions.

### Transcriptomic response correlates with tree co-occurrence

Combined with the observed natural spatial distributions of the 21 species and null modeling of species co-occurrence patterns, the transcriptomic information enabled us to quantify whether overall similarity in gene set enrichment in response to drought was non-randomly associated with natural tree species co-occurrence and to identify which individual molecular functions and biological processes were most strongly linked to natural tree species co-occurrence.

Next, we contrasted our gene expression analyses with analyses of the number of days to wilting in our drought experiment (Methods) as well as analyses of the functional trait and phylogenetic relatedness of the 21 species. In our previous work^18^, we have quantified eight commonly measured functional traits (leaf %C, %N, %P, wood density, leaf area, specific leaf area, maximum height, and seed mass) and have constructed a molecular phylogeny containing these 21 species^19^. Using the wilting, trait, and phylogenetic data, the spatial distributions of the 21 species in the forest and null models, we quantified whether similar species, based on wilting, functional traits or relatedness, non-randomly co-occurred. These results were then compared to the results from the analogous gene expression analyses.

We hypothesized that if drought is a key driver of species distributions, performance, and ultimately local co-occurrence, then species with similar overall gene set enrichment in response to experimental drought should naturally co-occur. Second, we hypothesized that those GO molecular functions and GO biological processes related to drought and desiccation will be strong predictors of natural species co-occurrence. Third, we hypothesized that transcriptomic similarity in response to drought would be most strongly linked with natural co-occurrence.

**Fig. 1:**
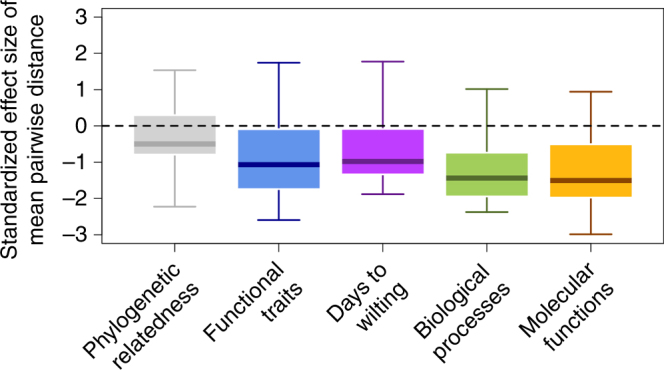
Species similarity and tree co-occurrence in a Wisconsin natural forest plot. The results from null modeling analyses for 630 20 × 20 m subplots where a standardized effect size of the mean pairwise distance is on the *y*-axis and the type of similarity is on the *x*-axis. Negative standardized effect size values indicate species were more similar than expected given a random expectation and positive values indicate species were more dissimilar than expected. A linear mixed effects model (*F* = 425.19; *p* < 0.01) with repeated measures and a Gaussian spatial covariance structure and subplot as a random effect followed by a Tukey Test showed that the boxplots were all significantly different from one another (*p* < 0.001)

Species with similar patterns of gene set enrichment in response to experimental drought across GO molecular functions and GO biological processes non-randomly co-occurred in nature (Fig. 1). This is indicated by the low standardized effect size (S.E.S.) values from the null model analyses. The result was robust to choice of the spatial scale of analysis, how species were clustered based upon the results from gene set enrichment analyses (Methods). Furthermore, we found little-to-no detectable phylogenetic signal in gene set enrichment (Tables 1, 2). In sum, similarity in the overall transcriptomic response between tree species to experimental drought is strongly linked to their co-occurrence in a natural stand. This indicates that differential transcriptomic responses to drought may be leading to differential distributions across hydraulic environments. The Wisconsin plot occurs on complex glacial outwash topography with wide variation in local substrate conditions, ranging from well-drained sandy loams on ridgetops and slopes to poorly-drained muck in depressions and drainage ways^20^. To investigate the local influence of response to drought in more detail, we mapped the distribution of S.E.S. values in the forest at the scale of 20 × 20 m for both GO biological processes and GO molecular functions. We correlated these maps with maps of volumetric soil water content using a Pearson’s correlation with a *p*-value estimated by a torus translation randomization^21^ that maintained the observed spatial auto correlation in the data. Soil water content was negatively correlated with both the GO biological process (*r* = 0.627; *p* < 0.005) and GO molecular function (*r* = 0.712; *p* < 0.005) S.E.S. maps (Table 3). In other words, more xeric soils in the forest plot are where co-occurring species are the most non-randomly similar in their gene set enrichment in response to experimental drought (Fig. 1). From this we infer that species with similar responses to drought may be partitioning soil hydraulic gradients in this forest.

**Table 1:** The biological process gene ontologies analyzed in this study

Gene ontology—biological process	Description	Mean standardized effect size of the mean pairwise distance	*D*	*p-*value
GO:0009414	Response to water deprivation	−1.36	1.28	0.771
GO:0009753	Response to jasmonic acid	−1.21	0.79	0.28
GO:0006979	Response to oxidative stress	−1.20	1.20	0.689
GO:0010200	Response to chitin	−1.17	1.10	0.574
GO:0009611	Response to wounding	−1.04	0.90	0.355
GO:0006629	Lipid metabolic process	−0.97	1.55	0.952
GO:0006855	Drug transmembrane transport	−0.97	0.85	0.306
GO:0016132	Brassinosteroid biosynthetic process	−0.93	1.58	0.951
GO:0000186	Activation of MAPKK activity	−0.91	1.29	0.758
GO:0006865	Amino-acid transport	−0.90	1.28	0.769
GO:0009737	Response to abscisic acid	−0.86	1.92	0.841
GO:0010363	Regulation of plant-type hypersensitive response	−0.78	1.49	0.941
GO:0006869	Lipid transport	−0.78	0.52	0.128
GO:0010075	Regulation of meristem growth	−0.76	0.75	0.268
GO:0006508	Proteolysis	−0.74	1.61	0.903
GO:0010103	Stomatal complex morphogenesis	−0.72	1.47	0.813
GO:0009750	Response to fructose	−0.72	1.23	0.669
GO:0015979	Photosynthesis	−0.69	1.69	0.942
GO:0006098	Pentose-phosphate shunt	−0.67	1.82	0.977
GO:0009744	Response to sucrose	−0.63	1.16	0.498
GO:0009860	Pollen tube growth	−0.62	1.48	0.72
GO:0015996	Chlorophyll catabolic process	−0.61	0.15	0.173
GO:0006633	Fatty acid biosynthetic process	−0.61	0.13	0.164
GO:0006598	Polyamine catabolic process	−0.60	−1.69	0.011
GO:0010025	Wax biosynthetic process	−0.60	2.16	0.876
GO:0006457	Protein folding	−0.58	1.82	0.805
GO:0009269	Response to desiccation	−0.55	1.73	0.804
GO:0009624	Response to nematode	−0.52	0.48	0.124
GO:0009987	Cellular process	−0.42	3.55	0.551
GO:0009615	Response to virus	−0.42	6.22	0.819
GO:0000226	Microtubule cytoskeleton organization	−0.41	4.46	0.795
GO:0009944	Polarity specification of adaxial/abaxial axis	−0.41	−6.44	<0.001
GO:0006816	Calcium ion transport	−0.41	4.00	0.606
GO:0009627	Systemic acquired resistance	−0.40	4.48	0.814
GO:0009058	Biosynthetic process	−0.40	7.22	0.908
GO:0006811	Ion transport	−0.39	5.46	0.766
GO:0019684	Photosynthesis, light reaction	−0.39	4.03	0.546
GO:0006099	Tricarboxylic acid cycle	−0.38	1.38	0.442
GO:0006810	Transport	−0.37	7.84	0.911
GO:0016556	mRNA modification	−0.37	6.56	0.914
GO:0006470	Protein dephosphorylation	−0.37	1.33	0.437
GO:0006412	Translation	−0.21	1.10	0.575
GO:0006857	Oligopeptide transport	−0.09	1.45	0.891
GO:0009409	Response to cold	0.00	1.60	0.971
GO:0006073	Cellular glucan metabolic process	0.00	1.22	0.71
GO:0008152	Metabolic process	0.33	1.51	0.705
GO:0016310	Phosphorylation	0.37	0.86	0.326
GO:0006950	Response to stress	0.42	−0.10	0.006
GO:0008272	Sulfate transport	0.43	0.94	0.385
GO:0009607	Response to biotic stimulus	0.49	0.73	0.253
GO:0009664	Plant-type cell wall organization	0.52	1.34	0.831
GO:0006351	Transcription, DNA-templated	0.73	−1.13	0.278
GO:0019252	Starch biosynthetic process	0.77	−1.37	0.278
GO:0005975	Carbohydrate metabolic process	0.83	0.06	0.015
GO:0006468	Protein phosphorylation	0.96	−0.44	<0.001
GO:0006952	Defense response	1.02	1.22	0.724
GO:0006355	Regulation of transcription, DNA-templated	1.04	1.05	0.508
GO:0006200	ATP catabolic process	1.44	1.04	0.462
GO:0015706	Nitrate transport	3.17	−5.04	0.265

The first two columns provide the gene ontology identifier and description. The third column provides the average of the standardized effect size of the mean pairwise distance values for 630 20 × 20 m subplots. Negative standardized effect size values indicate species with more similar than expected gene set enrichment in response to drought are naturally co-occurring. The fourth column presents the *D* statistic, where a value of 1 indicates phylogenetic signal in the expression data (i.e., consistent with Brownian motion evolution on the phylogeny) and values higher than 1 indicate increasingly labile evolution. The final column presents the *p*-value for the *D* statistic

**Table 2:** The molecular function gene ontologies analyzed in this study

Gene ontology—molecular function	Description	Mean standardized effect size of the mean pairwise distance	*D*	*p-*value
GO:0004568	Chitinase activity	−1.34	0.29	0.031
GO:0005507	Copper ion binding	−1.32	0.04	0.011
GO:0005215	Transporter activity	−1.11	1.23	0.698
GO:0003677	DNA binding	−1.10	1.28	0.739
GO:0004553	Hydrolase activity, hydrolyzing O-glycosyl compounds	−1.10	−0.56	<0.001
GO:0003700	Transcription factor activity, sequence-specific DNA binding	−1.07	0.88	0.343
GO:0008171	O-methyltransferase activity	−0.79	0.83	0.284
GO:0004190	Aspartic-type endopeptidase activity	−0.69	1.88	0.975
GO:0009055	Electron carrier activity	−0.69	1.50	0.881
GO:0005509	Calcium ion binding	−0.68	1.20	0.665
GO:0016491	Oxidoreductase activity	−0.67	2.04	0.992
GO:0003825	Alpha,alpha-trehalose-phosphate synthase (UDP-forming) activity	−0.66	1.58	0.907
GO:0016301	Kinase activity	−0.62	2.08	0.907
GO:0000166	Nucleotide binding	−0.60	1.35	0.615
GO:0015416	Organic phosphonate transmembrane-transporting ATPase activity	−0.60	2.04	0.891
GO:0008271	Secondary active sulfate transmembrane transporter activity	−0.59	0.91	0.382
GO:0008810	Cellulase activity	−0.58	0.16	0.142
GO:0004185	Serine-type carboxypeptidase activity	−0.57	0.73	0.302
GO:0005516	Calmodulin binding	−0.56	1.67	0.799
GO:0008289	Lipid binding	−0.56	−0.71	0.037
GO:0004871	Signal transducer activity	−0.42	5.18	0.798
GO:0016168	Chlorophyll binding	−0.42	4.56	0.82
GO:0005351	Sugar:proton symporter activity	−0.42	6.21	0.952
GO:0016758	Transferase activity, transferring hexosyl groups	−0.42	2.62	0.502
GO:0004650	Polygalacturonase activity	−0.41	−6.26	0.133
GO:0004872	Receptor activity	−0.41	4.80	0.722
GO:0008168	Methyltransferase activity	−0.41	−7.22	<0.001
GO:0009922	Fatty acid elongase activity	−0.41	4.63	0.807
GO:0003723	RNA binding	−0.41	−3.57	0.13
GO:0008422	Beta-glucosidase activity	−0.41	−1.43	0.334
GO:0004806	Triglyceride lipase activity	−0.40	4.72	0.607
GO:0005488	Binding	−0.40	3.34	0.537
GO:0005315	Inorganic phosphate transmembrane transporter activity	−0.39	3.69	0.496
GO:0015297	Antiporter activity	−0.39	6.94	0.92
GO:0003993	Acid phosphatase activity	−0.36	1.20	0.424
GO:0005524	ATP binding	0.49	1.54	0.95
GO:0004842	Ubiquitin-protein transferase activity	0.66	−1.83	0.29
GO:0004672	Protein kinase activity	0.68	0.50	0.115
GO:0015198	Oligopeptide transporter activity	0.77	−1.33	0.28
GO:0003899	DNA-directed RNA polymerase activity	0.84	−1.67	0.275
GO:0003735	Structural constituent of ribosome	0.90	1.08	0.551
GO:0004674	Protein serine/threonine kinase activity	0.97	0.54	0.115
GO:0004091	Carboxylic ester hydrolase activity	1.13	0.96	0.435
GO:0003824	Catalytic activity	1.43	−0.30	0.009
GO:0004180	Carboxypeptidase activity	3.34	−2.55	0.255

The first two columns provide the gene ontology identifier and description. The third column provides the average of the standardized effect size of the mean pairwise distance values for 630 20 × 20 m subplots. Negative standardized effect size values indicate species with more similar than expected gene set enrichment in response to drought are naturally co-occurring. The fourth column presents the *D* statistic where a value of 1 indicates phylogenetic signal in the expression data (i.e., consistent with Brownian motion evolution on the phylogeny) and values higher than 1 indicate increasingly labile evolution. The final column presents the *p*-value for the *D* statistic

**Table 3:** The relationship between community dispersion and soil water content in the forest dynamics plot using point soil core data

Type of dispersion	*r*
Phylogenetic relatedness	0.514*
Functional traits	0.424*
Days to wilting	0.612*
GO biological processes	0.627*
GO molecular functions	0.712*
Shade tolerance	0.193*
Water-logging tolerance	0.019
Drought tolerance	0.402*

The Pearson’s correlation between the S.E.S. of the MPD values for 20 × 20 m subplots for those subplots where soil cores were taken. Significance, *p* < 0.05 indicated with an asterisk, was assessed by generating a null distribution of expected Pearson’s *r* values using a torus translation

### Gene ontology terms associated with species co-occurrence

The exact GO biological processes and GO molecular functions that were most closely tied to patterns of species co-occurrence were considered next. The top GO biological process ontologies where gene set enrichment similarity was most strongly linked to tree co-occurrence were response to water deprivation (GO:0009414). The next two most important were response to jasmonic acid (GO:0009753) and response to oxidative stress (GO:0006979) (Table 1). While we caution against over-interpretation of these results given that the GO annotations were generated in model organisms^17^, we do note that these gene ontologies have established linkages with water stress^22–27^. For example, during water stress the closure of stomata and excess light cause the photosynthetic apparatus to become over-reduced leading to photoinhibition and oxidative stress^22^. Additionally, the interplay between jasmonic acid and ABA signaling has been functionally demonstrated in *Arabidopsis* species under drought^28,29^. Response to abscisic acid (GO:0009767) was the 11th ranked biological process GO in our results (Table 1), where species that had similar gene set enrichment for this GO tended to co-occur.

The top GO molecular functions where similar gene set enrichment was linked to species co-occurrence were chitinase activity (GO:0004568), copper ion binding (GO:0005507), and transporter activity (GO:0005215) (Table 2). As with the biological processes, the most important molecular functions linked to tree co-occurrence are linked with plant stress^30–32^. In sum, we were able to quantify the transcriptomic response of species to experimental drought and using this information we successfully identified the biological process and molecular function GO terms that were most strongly linked with natural tree co-occurrences.

### Transcriptomes outperform other predictors of co-occurrence

The above results suggest that water availability is a key driver of local tree species distributions and patterns of co-occurrence in this Wisconsin forest. Quantifying the dynamic response of plants to key environmental drivers provides important information not captured in more traditional functional trait-based studies of plant communities that have often focused on a few traits and not plastic responses. We compared the S.E.S. values from the gene set enrichment analyses to analogous S.E.S. values calculated using days to wilting data, functional trait similarity and phylogenetic relatedness. The results show that gene set enrichment S.E.S. values were significantly lower than those calculated from wilting, trait, or relatedness data (Fig. 1). Next, we correlated the wilting, trait, and phylogenetic S.E.S. values with soil water content as done with the expression data. While these S.E.S. values were significantly correlated with soil water content, the relationships were not as strong as those for the S.E.S. values from the expression data (Table 3; Supplementary Table 4). This demonstrates that our transcriptomic analyses of gene expression were better at detecting non-random spatial co-occurrence in a natural stand of trees than were analyses of functional traits and phylogenetic relatedness. Transcriptomic analyses were further able to identify the key biological processes and molecular functions underlying this co-occurrence.

**Fig. 2:**
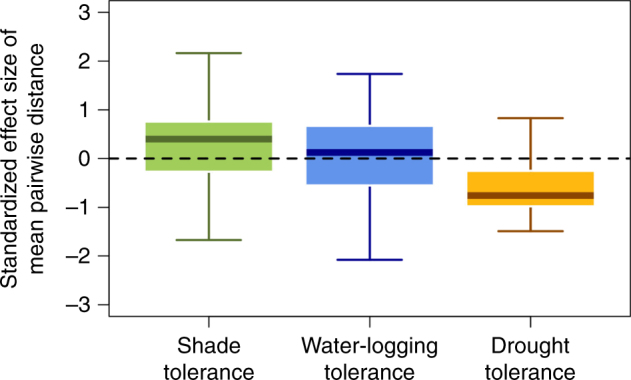
Environmental tolerance similarity and tree co-occurrence in a natural forest plot. The results from the analyses for 630 20 × 20 m subplots, where a standardized effect size of the mean pairwise distance is on the *y*-axis and the type of similarity is on the *x*-axis. Negative standardized effect size values indicate species were more similar in their tolerances than expected given a random expectation and positive values indicate species were more dissimilar than expected

Lastly, we considered the possibility that the species in our system spatially co-occurred due to their tolerances to other abiotic stressors that were not considered. For example, shade tolerance and water-logging tolerance are two additional important drivers of tree distributions^33^. Thus, we used a compilation of temperate tree shade, water-logging, and drought tolerance data from Niinemets and Valladares^33^ to address this possibility. Specifically, for each species we obtained their tolerances and calculated a similarity between species for each tolerance and conducted null modeling and soil water content correlative analyses just like we did for the expression, wilting, trait, and phylogenetic data (Methods). The results show that species with dissimilar shade and water-logging tolerances tend to co-occur and those with similar drought tolerances tend to co-occur (Fig. 2). Thus, tolerances to other factors are also significant drivers of tree distributions and co-occurrence in this forest. However, the effect sizes (S.E.S. values) are larger for drought tolerance, indicating that drought is a stronger predictor of tree co-occurrence in our study plot than the two other tolerances using an independent data source. We also note that the effect sizes from the expression data (Fig. 1) are larger than those generated from the drought tolerance data^33^ (Fig. 2), indicating that the expression data capture additional important information. Furthermore, while shade tolerance and drought tolerance S.E.S. values were correlated with soil water content, the strength of the correlations was weaker than that for expression S.E.S. values (Table 3; Supplementary Table 4).

## Discussion

Tree species distributions and co-occurrences are the result of the interaction between organismal function and environmental drivers, both of which are temporally dynamic. Here we have focused on the dynamic response of species to drought and its relationship to natural tree co-occurrence. Our focus on drought stems from previous research suggesting that drought governs species distributions across spatial scales^2,3^ and ecosystem flux^4,5^, and the impact of drought on forested ecosystems is expected to increase in frequency and intensity in the future^6^. We have presented a community transcriptomics-based approach that provides a broad assay of plant function and its dynamic response to changing conditions. The results show that species with similar transcriptomic responses to experimental drought tend to non-randomly co-occur along the soil water gradient in a natural forest stand. We were further able to identify the individual biological process and molecular function ontological domains most closely associated with natural co-occurrence. This analysis suggests that species with similar transcriptomic responses to decreasing water availability may have superior demographic performance, but future analyses that will leverage continued monitoring of this forest will be needed to test this directly. Our results also show that transcriptomic similarity was the strongest indicator of natural species co-occurrence compared to functional trait similarity and phylogenetic relatedness. Although measures of functional traits and phylogenetic similarity may provide useful initial insights into the drivers of community structure, a significant degree of important information is not captured using these measures. We do caution that the use of GO terms from distantly related model species is not a panacea and has weaknesses. As the technical, computational, and financial barriers to the use of transcriptomic approaches are lowered, particularly for non-model systems, trait-based community ecology will be poised to dramatically expand its capacity to broadly and dynamically assay functional diversity^34–36^. We expect that this will provide exciting new mechanistic insights into how phenotypes interact with their environments to drive community structure and dynamics now and into the future.

## Methods

### Forest dynamics plot and biodiversity data

This study aimed to quantify the linkages between gene expression similarity in response to experimental drought and the spatial co-occurrence of tree species in a natural community. To this end, we utilized data from the Wabikon Lake Forest Dynamics Plot—a Smithsonian ForestGeo Network site^37^. The Wabikon Lake plot is 25.2 hectares in area and is located in the Chequamegon-Nicolet National Forest in Wisconsin, USA. The forest plot is censused every 5 years, where all free-standing woody individuals ≥1 cm in diameter at 130 cm from the ground are identified, spatially mapped and have their diameters measured. The study used data from the 630 20 × 20 m subplots in the forest plot.

A soil survey of the plot was conducted in the summer of 2015, where soil volumetric moisture content was quantified by weighing fresh and dried soil cores. Soil cores were taken from the upper 10 cm of mineral soil using a regular 40 × 40 m grid inside the plot. Additionally, at every other 40 × 40 m point soil samples were taken at 2, 8, and 20 m from that point in a randomly chosen direction (Supplementary Fig. 3). This soil sampling design follows that of other forest dynamics plot in our research network^16^. Samples were taken over a 4-day period with no periods of rainfall during or immediately preceding the sampling period. In total, 252 samples were collected. A map of soil moisture content was generated by constructing spatial semivariograms and kriging at the resolution of 20 × 20 m such that a soil moisture value was estimated in each subplot.

This study used the first census of the plot performed in 2008 and focused on the 21 gymnosperm and angiosperm species that dominate the canopy (Supplementary Table 1). The only species that reaches a diameter >10 cm and has more than 100 individuals in the whole plot that was not considered in this study was *Fraxinus nigra* (Oleaceae). This species could not be sourced and grown in the greenhouse due to concerns regarding the spread of the Emerald Ash Borer (*Agrilus planipnnis*), which is currently decimating *Fraxinus* populations in the Upper Midwestern USA.

Our previous work in this forest has generated a molecular phylogeny and functional trait data for the species. Specifically, a three-locus barcode phylogenetic chronogram containing the species from this plot and 14 other ForestGeo plots was presented in Erickson et al.^19^ For this study, the Erickson et al. phylogeny was pruned to contain only the 21 study species. The functional trait data for this study comes from Swenson et al.^18^ in which foliar N, foliar P, specific leaf area, wood density, seed mass, leaf area, and maximum height were quantified for each species. Leaf nutrient and specific leaf area data were used to represent the well-documented leaf economics spectrum^38^, where species are arrayed along a spectrum of low investment and long leaf lifespans vs. high investment and short leaf lifespans. Wood density was used to represent the wood economics spectrum^39^, where species have low construction costs and mortality rates vs. high construction costs and mortality rates. Seed mass was used to represent a fundamental tradeoff between producing many poorly-provisioned offspring vs. a few well-provisioned offspring^40^. Leaf area was used to represent the organ level area for resource capture, which correlates with leaf temperatures^41^ and maximum height was used to estimate the adult light niche of the species^42^. In this study, as in our other work^18^, the trait data were used in a principle component (PC) analysis (Supplementary Table 2) to account for co-variation and the first two PC axes were used in the subsequent analyses of trait dispersion.

### Drought experiment

The drought experiment utilized 1-year old seedlings for all species. Seeds were originally sourced from the Upper Peninsula of Michigan and northern Wisconsin by Alpha Nurseries, Inc., Holland, Michigan within 200 km of the forest dynamics plot. While sampling from within the plot or immediately adjacent to the plot would have been ideal, it was not possible in the present student and likely introduces some bias. However, the geographic and climatic distance of collection sites from the forest plot is generally small indicating that local adaptation to dramatically different hydraulic environments was not likely. The relatedness of conspecific individuals is not known thereby limiting our ability to consider this degree of variation across species. However, multiple seeds were typically collected from a single adult individual indicating that there is likely a higher probability that the individuals studied are half-siblings. The seedlings were grown for one growing season prior to transplantation into the greenhouses at Michigan State University in the spring of 2014. After leaf emergence, the plants were grown for 8 weeks under well-watered conditions in the same room. Next, 10 plants of each species were selected for the drought treatment and the remaining plants continued under the same watering regime and were used as controls. The plants in the drought treatment no longer received water.

A LiCor-6400 was used to quantify leaf stomatal conductance and photosynthetic rate on control and droughted plants. Species-level distributions of stomatal conductance and photosynthetic rates were generated from control plants that could be compared to individual plants in the drought treatment. For control plants, immediately following LiCor measurements the same leaf was flash frozen in liquid nitrogen and transferred to a −80 °C freezer. Plants in the drought treatment were monitored daily for signs of wilt. If the plant appeared wilted, then its stomatal conductance and photosynthetic rate were quantified. If the values were >2 standard deviations from the mean of the control distribution for that species, leaves were flash frozen and placed in the freezer. The number of days to wilt per individual per species was recorded and an average days to wilting, and therefore days to tissue sampling, was recorded. For gymnosperms, where there was no visual wilting, a random individual was selected every 1 day for photosynthesis measurements. If that individual was significantly droughted, the other individuals from that species in the drought experiment were also measured and flash froze. Thus, the days to wilting for these species is the number of days from the start of the experiment to sampling and we refer to this as days to wilting in the paper for simplicity. The average difference in the days to wilting between species was < 20 days. Thus, there were no major ontogenetic changes between individuals of species wilting early in the study and those wilting later in the study and therefore biases due to ontogeny were not a concern, whereas they may have been a concern if our study organisms had shorter life cycles.

### RNA sequencing and expression analyses

A reference transcriptome to which we could map expression data was generated for each species. The reference for each species was generated using four control individuals and three droughted individuals. For all samples, RNA was extracted using an RNeasy Plant Mini Kit (Qiagen; Valencia, California) with RNA quality and quantity measured using a NanoDrop 2000 spectrophotometer and an Agilent Bioanalyzer 2100. RNAseq was performed at the Beijing Genomics Institute, Shenzhen, China using Illumina 2000 HiSeq platform using 100 bp paired-end reads and with eight samples per lane. Thus, there were ultimately seven samples used per species (4 control and 3 drought) and all samples were sequenced on the sample platform and at the same depth. The average number of clean reads per sample across all species was 60 million with a range of 51–68 million reads (Supplementary Table 1).

Reference transcriptomes were assembled de novo using Trinity^43^. The N50 values for the species assemblies ranged from 1291 to 1835 (Supplementary Table 1). References were annotated against the NCBI nr and SwissProt databases using blastx where only the top hit was retained if multiple significant hits (*E*-values < 1 × 10^−5^) were detected and Blast2GO^44^ was utilized to assign GO terms for biological processes and molecular functions. SOAPaligner^45^ was used to map reads from the four control and three drought samples per species to their respective reference transcriptomes with no more than five mismatched bases allowed for mapping. The counts data were then used in edgeR^46^ to quantify reads per kilobase of transcript per million mapped reads (RPKM) for the two groups (control and drought) using all samples from the group. We then used edgeR to quantify differential expression (log_2_ fold changes of ±1 were used as cutoffs) and adjusted *p*-values using a Benjamini–Hochberg false discovery rate.

Next, genes were grouped by GO terms that were found across all 21 species in the experiment. In other words, if a GO term was found in less than all 21 species it was not included in any downstream analyses. Thus, there was GO information for a particular species not found in all other species and therefore could not be readily compare. For example, *Acer saccharum* had more than 1000 GO terms identified for biological processes and molecular functions, but fewer than 100 of these could be compared across all species (Tables 1, 2). Future work taking a functional phylogenomic approach that can construct individual gene trees or that looks at GO terms where only some species are represented should be conducted and may prove informative. However, that is beyond the scope of the present study and the following statistical tests and the false discovery rate adjustments were based upon only those GO terms found in all 21 species. A Fisher’s exact test was then used to assess gene set enrichment for each GO term again using a Benjamini–Yekutieli false discovery rate adjustment^47^. The odds ratio was then clustered across species using hierarchical clustering (Supplementary Figs. 1, 2). Similarly, species were hierarchically clustered based upon whether they were significantly enriched or not (Supplementary Figs. 1, 2). The branch lengths from these hierarchical clustering dendograms using the significance data were used for downstream analyses of similarity and natural co-occurrence where species with less branch length separating them had more similar overall gene set enrichment in response to experimental drought than did species with more branch length separating them. Subsequent analyses also investigated similarity using individual GO terms where similarity between species was based upon whether they had the same (significant–significant or non-significant–non-significant) or different (significant–non-significant or non-significant–significant) gene set enrichment in response to experimental drought.

### Community dispersion analyses

We quantified whether tree species in the Wabikon Lake forest plot non-randomly co-occurred with respect to phylogenetic relatedness, functional trait similarity, environmental tolerance similarity^33^, days to wilting similarity, and similarity in gene set enrichment in response to experimental drought using biological process and molecular function GO categories. The environmental tolerance data were taken from Niinemets and Valladares^33^ who estimated the relative tolerance of temperate tree species, inclusive of our study species, to drought, shade, and water-logging. The rationale for using this data was that it provided an alternative and independent assay of drought tolerance and allowed us to investigate if other axes of environmental tolerance (i.e., shade and water-logging) were as or more important in our study system. The days to wilting data were used independently of the functional trait data as a separate and experimental assay of drought tolerance. All analyses used a mean pairwise distance (MPD) between all individual trees in a sample as:

$${\rm{MPD}} = \frac{{\mathop {\sum }\nolimits_i^n \mathop {\sum }\nolimits_j^n \delta _{i,j}f_if_j}}{{\mathop {\sum }\nolimits_i^n \mathop {\sum }\nolimits_j^n f_if_j}},$$

where *f*_*i*_ is the number of individuals of species *i*, *f*_*j*_ is the number of individuals of species *j*, and *δ*_*i,j*_ is the phylogenetic distance between two taxa, the Euclidean distance between two taxa positioned on the two PC axes derived from the functional trait, tolerance or days to wilting data, the distance between two species on the hierarchical clustering of gene set enrichment for all GO molecular functions (Supplementary Fig. 1) or the distance between two species on the hierarchical clustering of gene set enrichment for all GO biological processes (Supplementary Fig. 2). These observed values were then compared to a null distribution of MPD values generated by randomizing the names of taxa on the respective data sets 999 times and a S.E.S. was calculated as:

$$\rm{S.E.S.} = \frac{{MPD_{obs} - meanMPD_{null}}}{{st.devMPD_{null}}},$$

where MPD_obs_ is the observed MPD value, meanMPD_null_ is the mean MPD value in the null distribution, and st.devMPD_null_ is the standard deviation of the null distribution^48^. A negative S.E.S. value indicated co-occurring species were more similar than expected and a positive value indicated co-occurring species were less similar than expected. The analyses were conducted using 20 × 20 m subplots, but similar results at the 40 × 40 m and 100 × 100 m scales were found. We show only the 20 × 20 m results because they were consistent with other scales and because our elevation data set was collected at this scale making our downstream spatial analyses at this scale possible.

Next, we performed the above analyses using a single GO biological process or molecular function category at a time (i.e., a single column in Supplementary Figs. 1 and 2). In these analyses the *δ*_*i,j*_ value in the MPD calculation was 0 if the two species both had significant or non-significant gene set enrichment for that category and 1 if one species had a significant enrichment while the other did not. As with the above analyses, a null model was implemented and a S.E.S. value was calculated. We then ranked GO categories based on their average S.E.S. value across all 20 × 20 m subplots to determine which individual biological processes and molecular functions were those where similarity in gene set enrichment was most strongly related to tree co-occurrence.

### Spatial analyses

To test the expectation that similarity in gene expression response to experimental drought would be most evident in natural species assemblages on more xeric edaphic environments, we utilized the soil water content data from the 252 soil cores taken in the plot. Specifically, we correlated the S.E.S. values for the phylogenetic, functional trait, days to wilting, tolerances, GO biological processes, and the GO molecular functions from those 20 × 20 m subplots that had soil cores taken with the soil water content data and used a torus translation that accounts for spatial autocorrelation to quantify a *p*-value^21^. In a secondary analysis, we estimated the soil water content in each of the 630 20 × 20 m subplots using kriging. The kriged values were then correlated with the S.E.S. values and *p*-values were again assessed using a torus translation.

### Phylogenetic signal analyses

A concern regarding our approach and arising from our results could be that a strong phylogenetic signal in the data may be biasing our results and our interpretations. For example, if gymnosperms tend to cluster together in their gene expression responses, in general or for specific GO terms, and tend to co-occur in the forest plot for reasons not linked to drought response, we may have flawed inferences regarding the importance of gene expression response to drought. Thus, if there is phylogenetic signal in the data, a phylogenetic comparative method should be used. We quantified the degree of phylogenetic signal in the expression data in two different ways. First, to quantify the phylogenetic signal in the overall similarity in gene set enrichment we utilized a Mantel test relating phylogenetic distance to the distance between two species on the gene set enrichment hierarchical clustering dendrograms used for our dispersion analyses (Supplementary Figs. 1, 2). If there was positive and significant relationship, then this would be evidence of phylogenetic signal.

Our second approach investigated individual GO terms. For this analysis, as in our dispersion analyses, species were coded as having a significant gene set enrichment for a specific GO term (1) or no significant enrichment (0). Thus, the coding of species was binary. Phylogenetic signal in binary data is best measured using the *D* statistic of Fritz and Purvis^49^ where Brownian Motion trait evolution is where *D* = 1, labile trait evolution is where *D* > 1, and trait evolution more conservative than Brownian Motion is where *D* < 1. The significant of the *D*-value is gauged using a permutation test, where observed values are randomized across the tips of the phylogeny 999 times and a null distribution of *D*-values is estimated.

### Data availability

Data are available in Dryad (10.5061/dryad.q3j60) and NCBI-SRA (PRJNA413418). The authors declare that all other data supporting the findings of this study are available from the corresponding authors upon request.

## Electronic supplementary material

Supplementary Information
