# Supplementary material for: Tree co-occurrence and transcriptomic response to drought
Source: Nat Commun. 2017 Dec 8;8:1996. doi: 10.1038/s41467-017-02034-w (PMC5722877; doi:10.1038/s41467-017-02034-w)
Supplement: Supplementary file 1 — Supplementary Information [file 41467_2017_2034_MOESM1_ESM.pdf]

Supplementary Table 1. The 21 gymnosperm and angiosperm tree species in this study that dominate the canopy of the Wisconsin forest plot analyzed The family, genus and species are given as well as two indicators of relative species dominance. Specifically, for each species the percent of all individual trees >5cm in diameter and the percent of the stem basal area for all individual trees >5 cm in diameter are given.

Family	Genus	Species	Species code	Percent of trees in plot > 5cm in diameter	Percent of basal area in plot for trees > 5cm in diameter	N50 Value from *de novo* assembly of Unigenes (bp)	Average number of reads per sample (rounded to nearest million)
Pinaceae	Picea	glauca	PIG	5.545	4.232	1291	51
Pinaceae	Larix	laricina	LL	0.015	0.200	1664	58
Pinaceae	Abies	balsamea	AB	5.384	1.0731	1787	57
Pinaceae	Tsuga	canadensis	TC	0.054	0.048	1835	63
Cupressaceae	Thuja	occidentalis	TO	0.576	0.568	1832	62
Cornaceae	Cornus	alternifolia	CA	0.020	0.002	1531	57
Oleaceae	Fraxinus	americana	FA	5.872	10.394	1520	52
Malvaceae	Tilia	americana	TA	9.507	22.646	1460	59
Sapindaceae	Acer	saccharum	ASII	40.442	39.684	1694	61
Sapindaceae	Acer	rubrum	AR	0.767	0.554	1728	55
Sapindaceae	Acer	spicatum	AS	0.049	0.004	1773	60
Salicaceae	Populus	tremuloides	PT	12.008	6.259	1737	64
Salicaceae	Populus	grandidentata	POG	2.697	1.804	1583	60
Salicaceae	Populus	balsamifera	PB	0.005	0.006	1524	59
Betulaceae	Alnus	incana	AI	0.044	0.004	1632	56
Betulaceae	Betula	papyrifera	BP	0.816	0.886	1564	58
Juglandaceae	Juglans	cinerea	JC	0.083	0.281	1599	59
Ulmaceae	Ulmus	americana	UA	0.244	0.069	1471	57
Rosaceae	Prunus	serotina	PRS	0.357	0.150	1689	67
Rosaceae	Prunus	virginiana	PV	0.073	0.006	1634	61
Rosaceae	Amelanchier	laevis	AL	0.015	0.003	1544	65
**Total**				84.573	88.873		

Supplementary Table 2. The loadings from a principle component (PC) analysis of the 8 functional traits measured in this study A PC analysis was used to mitigate redundancy in the functional trait data prior to functional dispersion analyses. Data were scaled and if where necessary data were log-transformed to approximate normality. The first component explained 47.062% of the variance and the second component explained 17.036% of the variance. The Euclidean distance between species on these two PC axes was used to generate a functional trait distance matrix used to calculate community mean pairwise distances.

Trait	Component 1 Loading	Component 2 Loading
Leaf %P	-0.272	0.606
Leaf %N	-0.400	0.120
Leaf %C	0.425	0.001
Wood Density	-0.290	-0.410
Leaf Area	-0.459	0.125
Specific Leaf Area	-0.436	-0.107
Maximum Height	0.168	0.650
Seed Mass	-0.269	0.017

Supplementary Table 3. The number of expressed genes per species and the number differentially expressed The data were analyzed using the software package edgeR[40] and we report the total number of genes expressed as well as the number of genes over-expressed (up) or under-expressed (down) in the drought treatment compared to the control treatment. Not all expressed genes could be assigned to a GO category and not all GO categories were found in all species. So not all expressed genes reported here were used in the study.

Genus	Species	Number of Differentially Expressed Genes (up)	Number of Differentially Expressed Genes (down)	Total Number of Genes Expressed
Picea	*glauca*	2608	2318	49240
Larix	*laricina*	2191	3229	71907
Abies	*balsamea*	4160	2025	58732
Tsuga	*canadensis*	3872	3574	65922
Thuja	*occidentalis*	11825	7757	78072
Cornus	*alternifolia*	6448	2932	73343
Fraxinus	*americana*	1227	4673	58706
Tilia	*americana*	7380	9107	68451
Acer	*saccharum*	4991	5043	48362
Acer	*rubrum*	6015	8585	59202
Acer	*spicatum*	5802	1709	53796
Populus	*tremuloides*	5511	3946	68983
Populus	*grandidentata*	2042	7753	60445
Populus	*balsamifera*	5055	6790	54879
Alnus	*incana*	3693	2984	59894
Betula	*papyrifera*	6079	6139	64078
Juglans	*cinerea*	8830	8461	68171
Ulmus	*americana*	5866	4436	45571
Prunus	*serotina*	2596	3723	58672
Prunus	*virginiana*	7852	6827	61185
Amelanchier	*laevis*	7218	5420	69835

Supplementary Table 4. The relationship between community dispersion and soil water content in the forest dynamics plot The Pearson's correlation between the standardized effect size (S.E.S.) of the mean pairwise distance (M.P.D.) values for 630 20 × 20m subplots and the soil water content. Significance, $P < 0.05$ indicated with an asterisk, was assessed by generating a null distribution of expected Pearson's *r* values using a torus translation. We assessed whether the strength of the phylogenetic or trait correlations was significantly weaker than the GO correlations using a Fisher r-to-z transformation. In all cases, the phylogenetic and trait correlations were significantly weaker than the GO correlations.

**Type of Dispersion**	***r***
Phylogenetic Relatedness	0.604*
Functional Traits	0.564*
Days to Wilting	0.609*
GO Biological Processes	0.665*
GO Molecular Functions	0.711*
Shade Tolerance	0.206*
Water Logging Tolerance	0.028
Drought Tolerance	0.372*

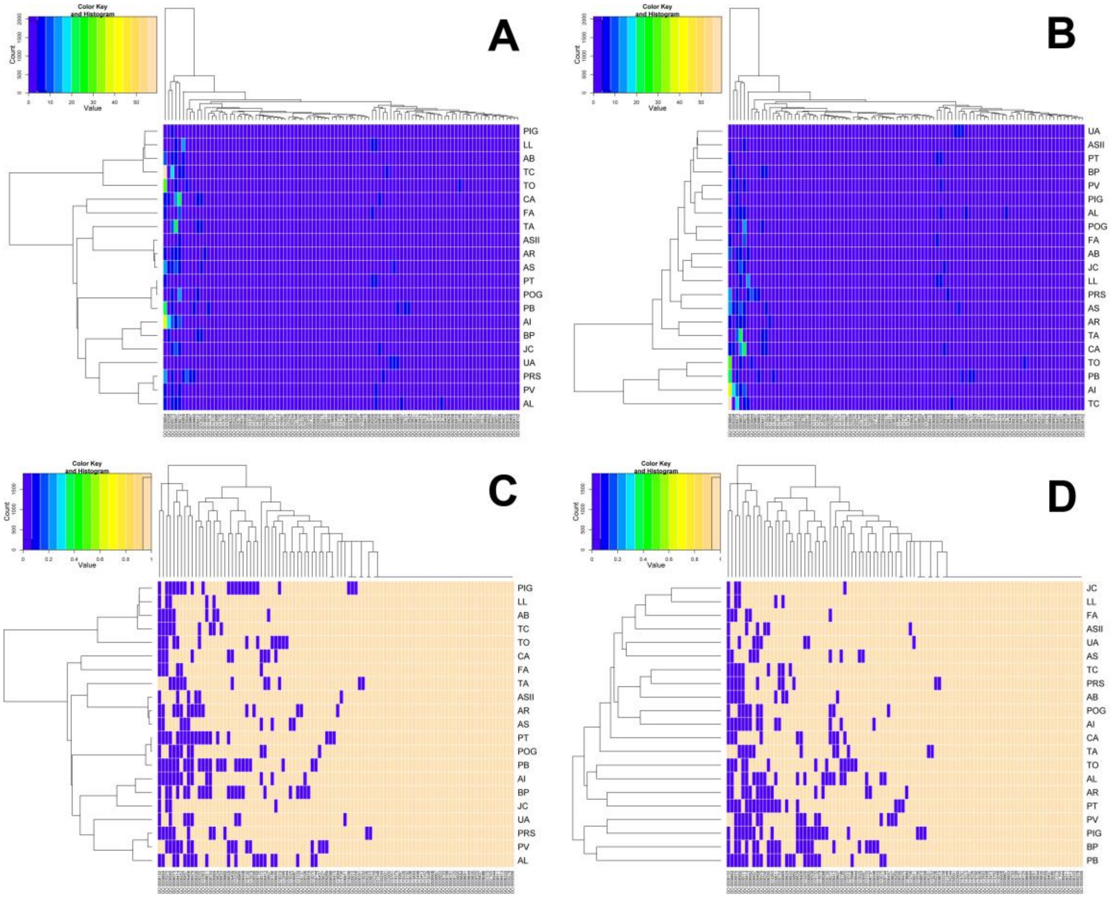

Supplementary Figure 1. Clustering analysis of GO biological process gene set enrichment in response to experimental drought The odds ratio values from a Fisher's Exact Test for gene set enrichment are presented where columns indicate individual GO categories organized by the hierarchical clustering dendrogram at the top of the matrix. Warm colors indicate a higher ratio (i.e. larger change). The rows in panel A are ordered by a phylogenetic tree on the left of the matrix. The rows in panel B are ordered by a hierarchical clustering diagram to the left of the matrix. Panels C and D display whether the Fisher Exact Test was significant (purple) or not (pink) after a Benjamini-Yekutieli correction with rows clustered by phylogeny (panel C) or hierarchical clustering (panel D). The hierarchical clustering dendrogram to the left of the matrix in panel D was used for the biological process analyses presented in the main text, which were comparable to analyses using the dendrogram to the left of the matrix in panel B. In the upper left of each panel is a dendrogram displaying the distribution of values in the matrix for that panel. A Mantel Test for phylognetic signal in the data using the dendrograms on the roles of panel's C and D was not significant (p >0.05). Codes for species names (*see* Extended Data Table 1) are given to the right of the panel in each matrix.

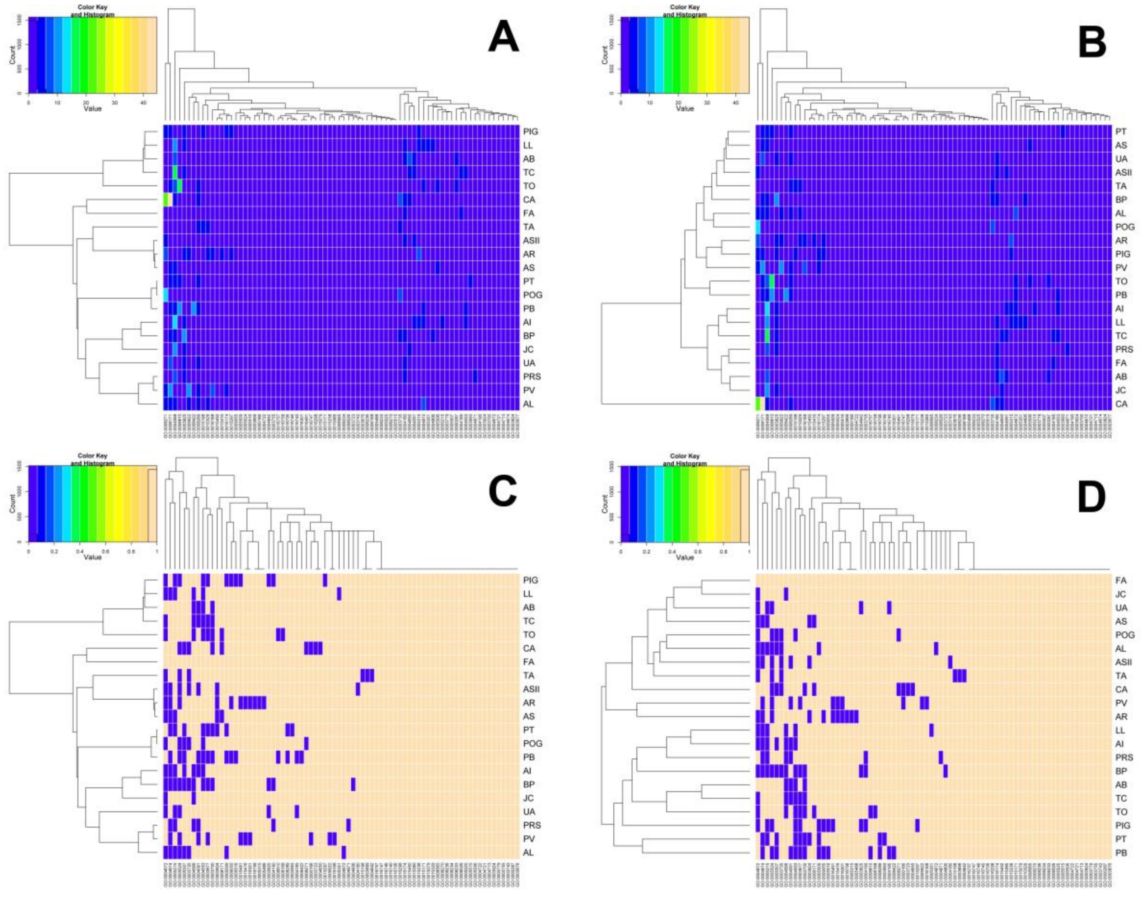

Supplementary Figure 2. Clustering analysis of GO molecular function gene set enrichment in response to experimental drought The odds ratio values from a Fisher's Exact Test for gene set enrichment are presented where columns indicate individual GO categories organized by the hierarchical clustering dendrogram at the top of the matrix. Warm colors indicate a higher ratio (i.e. larger change). The rows in panel A are ordered by a phylogenetic tree on the left of the matrix. The rows in panel B are ordered by a hierarchical clustering diagram to the left of the matrix. Panels C and D display whether the Fisher Exact Test was significant (purple) or not (pink) after a Benjamini-Yekutieli correction with rows clustered by phylogeny (panel C) or hierarchical clustering (panel D). The hierarchical clustering dendrogram to the left of the matrix in panel D was used for the molecular function analyses presented in the main text, which were comparable to analyses using the dendrogram to the left of the matrix in panel B. In the upper left of each panel is a dendrogram displaying the distribution of values in the matrix for that panel. A Mantel Test for phylognetic signal in the data using the dendrograms on the roles of panel's C and D was not significant ($p > 0.05$). Codes for species names (*see* Extended Data Table 1) are given to the right of the panel in each matrix.

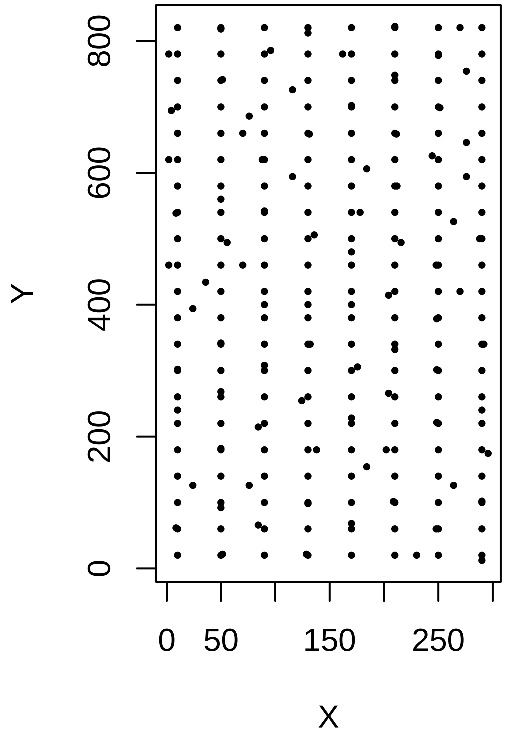

Supplementary Figure 3. The spatial distribution of soil cores taken inside the forest dynamics plot The black points represent the samples from the regular 40x40m grid and the additional points.
